# Effect of Size and Location of Nevi on Postoperative Pain and Emergence Agitation in Children Undergoing Nevi Excision

**DOI:** 10.3390/jcm8010106

**Published:** 2019-01-17

**Authors:** Jin-Soo Kim, Hye Sun Lee, Dong Ha Park, Suhyun Seok, Tae Kwang Kim, Hye Seon Lee, Ji Eun Kim

**Affiliations:** 1 Department of Anesthesiology and Pain Medicine, Ajou University School of Medicine, Suwon 164, Korea; jskane@aumc.ac.kr (J.-S.K.); tjgtngus12@gmail.com (S.S.); itsagoodtimee@gmail.com (T.K.K.); alredy@naver.com (H.S.L.); 2 Biostatistics Collaboration Unit, Yonsei University College of Medicine, Seoul 03722, Korea; HSLEE1@yuhs.ac; 3 Department of Plastic and Reconstructive Surgery, Ajou University School of Medicine, Suwon 164, Korea; growhand@aumc.ac.kr

**Keywords:** child, emergence agitation, nevi, pain

## Abstract

Congenital melanocytic nevi need surgical excisions. However, the effect of the size and location of the nevi on pain and emergence agitation have yet to be studied. The objective of this study was to evaluate (1) the ideal parameter of the nevus size and (2) the effects of the size and location of the nevus on pain and emergence agitation. This observational study enrolled 100 children scheduled for an excision of a nevus under sevoflurane anesthesia. The parameters of the nevus size included the long diameter, the area before resection, the area of resection, and the proportion (the area of resection/total body surface). The nevus locations included the trunk, face, scalp, and extremities. The proportion of the nevi was the most ideal parameter in evaluating the pain and emergence agitation. A large size showed a higher emergence agitation than a small size (median (range); 6 (0–20) in small groups vs. 12.5 (0–20) in large groups, *p* = 0.021). However, the pain was comparable. The nevus location did not influence pain or emergence agitation. In a multivariate regression analysis, a younger age and an extensive excision were associated with higher pain and emergence agitation. In conclusion, large nevi induced more severe emergence agitation. However, the nevus location did not affect the outcome. In addition, a younger age was associated with pain and emergence agitation. Clinicians need to consider the proportion of nevi when managing children undergoing a nevus excision.

## 1. Introduction

Congenital melanocytic nevi (CMN) are defined as benign melanocytic nevi that are present at birth. CMN is found in 1% to 6% of neonates. However, nevi being identical CMN are reported to show a prevalence of more than 15% in older children [1]. CMN tend to extend deeper into the dermis and subcutaneous tissues compared with acquired melanocytic nevi, and large CMN are associated with a higher risk for melanoma than smaller CMN, although the exact risk is unknown. Because of the potential for malignant changes, the enlargement in proportion to the child’s growth, and the cosmetic appearance, prophylactic surgical excision is usually advocated early in life, although there is no consensus about the risk of melanoma and optimal management practices in CMN [2].

Effective pain management in children remains a challenge. A large proportion of children are reported to receive insufficient analgesic medication after surgery [3], and thus, suffer from moderate-to-severe postoperative pain up to 44% [4]. Skin incisions alone, similar to a nevus excision, is sufficient to induce mechanical or heat hyperalgesia, although cutaneous combined with deep tissue incisions trigger prolonged hyperalgesia [5]. In addition, sensitivity to painful stimuli is reported to be affected by the specific body region [6]. Although disputed, the sensitivity in the facial area was higher compared with that in other body regions.

The emergence agitation in children occurs generally during recovery from sevoflurane anesthesia. Although the emergence agitation resolves spontaneously, it increases the risk of patient injury, surgical site damage, and parental dissatisfaction with any extra treatments. Emergence agitation has been attributed to multiple factors including age, temperament, pain, rapid anesthetic emergence, and anesthetic and surgical types.

Turkmen et al. [7] compared the CMNs based on dimension, total area, and total area-to-body surface area and proposed the total nevus area for determining the risk of a malignant transformation. However, no reliable parameter based on nevus size is available for estimation of the postoperative pain and emergence agitation after a nevus excision. In addition, no studies have investigated the association between the size or body region of a nevus and the degrees of pain and emergence agitation.

Therefore, the aims of this study were to evaluate (1) the adequate parameters of the nevus size and (2) the effects of the size and location of the nevus on postoperative pain and emergence agitation in preschool children undergoing a nevus excision.

## 2. Materials and Methods

This single-center prospective observational study was approved by the Ajou University Hospital Institutional Review Board (protocol number: AJIRB-MED-OBS-16-533) and was registered at http://clinicaltrials.gov (registration number: NCT03094754). Between March 2017 and January 2018, 100 children who were scheduled to undergo general anesthesia for the excision of nevi were enrolled. All parents provided written informed consent. The inclusion criteria were the American Society of Anesthesiologists physical status 1 and 2, the age between 1 and 7 years, previously healthy or with mild systemic disease, an excision warranting general anesthesia, and an elective surgery. The exclusion criteria were developmental, psychiatric, and neuromuscular diseases; an excision of nevi involving more than 3 sites, and giant nevi.

All children had intravenous (IV) access and were not treated with any medication previously. On arrival at the operating room, standard monitors including an electrocardiogram, pulse oximeter, and application of noninvasive blood pressure were used. Anesthesia was induced with 5 mg/kg of thiopental and 0.3 µg/kg of fentanyl. After confirming the absence of a response to eye stimulus, 0.6 mg/kg of rocuronium was administered. Ventilation was conducted with a mask using 100% oxygen and sevoflurane 5 vol%, followed by orotracheal intubation. Anesthesia was maintained with the end-tidal concentration of sevoflurane 2.0–2.5 vol% during surgery. We did not perform the regional block or premedication. All lesions were excised totally or in stages, together with the underlying subcutaneous tissue, and with a tension-free closure, regardless of melanoma suspicion. Surgical methods comprised the direct primary closure and excision with a local flap but did not include the excision with a skin graft, the use of tissue expander, and a dermabrasion. After the surgery, the children were extubated after a neuromuscular reversal and transferred to the postanesthesia care unit (PACU). If the Modified Aldrete score was ≥8, children were transferred to a ward.

The parameters related to the size of the nevi included the long diameter, the area before resection, the area of resection, and the proportion. The long diameter was measured in centimeters using a ruler. The area was measured by tracing the total nevus area onto a transparent film, Opsite Flexigrid (Smith & Nephew, Inc., Hull, UK); to accurately assess the size of the nevus in cm^2^, the tracing was transposed onto paper divided into millimeters, which is considered to be accurate up to 1 mm. The “area before resection” was measured at the time between the anesthesia induction and the start of the surgery. The “area after resection” was measured at the time between the end of surgery and the surgical dressing by adding a semipermeable polyurethane membrane (Tegaderm^®^) under the transparent film. The “area of resection” was calculated as the area before resection minus the area after resection. The total body surface (TBS) area was estimated for each patient using the formula of TBS (cm^2^) = 0.007184 × weight (kg)^0.425^ × height (cm)^0.725^. The proportion as area of resection/TBS area was calculated as a percentage. The regions of the nevi were defined as the trunk, face, scalp, and extremities. Postoperative pain was assessed using the Face, Legs, Activity, Cry, Consolidation (FLACC) scale (0 = no pain, 1–3 = mild discomfort, 4–6 = moderate pain, and 7–10 = severe discomfort or pain), which is a measurement tool used to assess pain among children between the ages of 2 months and 7 years or in individuals who are unable to communicate their pain. The emergence agitation was rated with the Pediatric Agitation and Emergence Delirium (PAED) scale. Children were defined as having pain if the FALCC score was ≥4 or emergence agitation if the PAED score was ≥10. Pain and emergence agitation was evaluated upon PACU arrival, at 10 min, 30 min, 1 h, and 2 h after surgery. Fentanyl 0.5 µg/kg was administered in patients with pain ≥4 on the FLACC scale. Hemodynamic data was recorded before induction (baseline), after intubation, at the start of surgery, at the end of surgery, and after extubation. A single anesthesiologist observed and assessed the children throughout the study. For analysis, the children were divided into 2 groups according to the median value of proportion: a small group with less than 0.042 cm in proportion (*n* = 50) and a large group as 0.042 or greater in proportion (*n* = 50).

The results were expressed as the mean ± standard deviation, the median (range), or the number of patients (%). The normality of the distribution was assessed with the Shapiro–Wilk test and the parametric and nonparametric data were analyzed using the independent *t*-test and the Mann–Whitney *U*-test, respectively. Categorical variables were evaluated by the chi-square test or Fisher’s exact test when appropriate. Repeatedly, measured data were analyzed with a linear mixed model. A multivariate linear regression analysis was performed to assess the independent factors predicting pain and agitation. A value of *p* <0.05 was considered statistically significant. The statistical analyses were conducted using IBM SPSS Statistics ver. 23.0 (IBM Corp., Armonk, NY, USA).

## 3. Results

A total of 100 patients completed the study. The parameters for the measurement of the nevus size and location are shown in Table 1. The proportion defined as the ratio of nevi to TBS was the most representative of the postoperative pain and emergence agitation compared with the long diameter, the area before resection, and the area of resection. The location of the nevus did not influence pain and emergence agitation.

**Figure 1:**
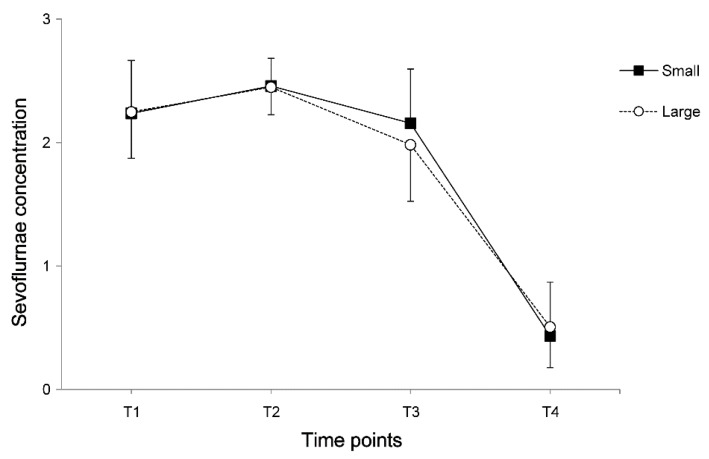
The intraoperative concentration of sevoflurane. Data are expressed as the mean ± standard deviation: T1, after anesthesia induction; T2, the start of surgery; T3, the end of surgery; and T4, before extubation.

**Figure 2:**
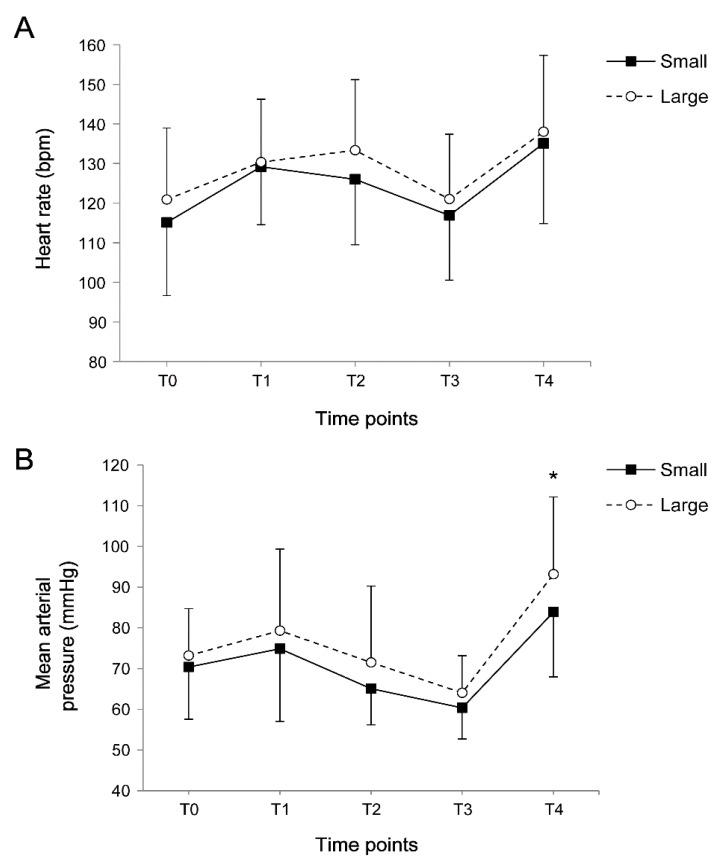
The (**A**) heart rate and (**B**) mean arterial pressure during the perioperative period. Data are expressed as the mean ± standard deviation: T0, before anesthesia induction (baseline); T1, after intubation; T2, the start of surgery; T3, the end of surgery; and T4, after extubation.

Among the 100 children, the incidence of emergence agitation was 51% and the incidence of pain was 66%. Classification into two groups according to the median value of proportion showed that the patient’s characteristics were comparable between the two groups (Table 2). However, the operation time, anesthesia time, and nevus size were significantly higher in the large group. The intraoperative end-tidal concentration of sevoflurane was constantly maintained during surgery within 2.5 vol%, without any significant differences between the two groups (Figure 1). Perioperative heart rate was similar; however, mean arterial pressure after extubation was higher in the large group compared with the small group (*p* = 0.007, Figure 2).

Table 3 lists the postoperative recovery profiles. For the highest pain score during postoperative 2, HR did not differ between the two groups and the number of children receiving the analgesic for pain treatment was similar. However, the highest emergence agitation score was significantly higher in the large group (median values; 6 in small group vs. 12.5 in large groups, *p* = 0.021). The duration of the PACU stay was comparable between the two groups.

To investigate the predictive factors related to pain and emergence agitation, a multivariate linear analysis was performed (Table 4). With regards to pain, a younger age had higher pain (*β* (standard error) = −0.049 (0.014), *p* = 0.001) and a high proportion had higher pain (*β* (SE) = 7.913 (2.709), *p* = 0.004). With regards to emergence agitation, a younger age had higher pain (*β* (SE) = −0.097 (0.031), *p* = 0.002) and a high proportion had higher pain (*β* (SE) = 15.803 (6.061), *p* = 0.011).

## 4. Discussion

The primary endpoints included the effects of the size and location of the nevus on postoperative pain and emergence agitation. The proportion was the most ideal parameter when defining the role of the nevus size in pain and emergence agitation. The large-sized group was significantly associated with a higher emergence agitation following the sevoflurane anesthesia compared with the small-sized group. However, the postoperative pain was comparable between the two groups. The specific location of the nevus excision had no role in the differences in pain and emergence agitation associated with other areas of the body. In the multivariate linear regression analysis, a younger age and a large-sized excision were associated with increased pain and emergence agitation.

Emergence agitation is a postoperative behavioral disturbance observed after anesthesia in children, with a reported incidence of 70% following sevoflurane anesthesia [8]. Despite unclear mechanisms, sevoflurane may exert an irritable effect on the central nervous system [9]. Several possible risk factors of emergence agitation were observed in this study. First, the large nevi triggered a higher emergence agitation, although postoperative pain was comparable between large and small groups. Prolonged anesthesia time in the large group may increase the exposure to sevoflurane (55 min and 67.5 min in small and large groups, respectively), which may affect the development of emergence agitation. The relationship between postoperative pain and agitation is still disputed. Emergence agitation was found in 50% of children undergoing magnetic resonance imaging under sevoflurane anesthesia without surgery [10]. The elimination of postoperative pain via a scalp nerve block failed to reduce the occurrence of emergence agitation following sevoflurane anesthesia in children undergoing an excision of scalp nevi [11]. Conversely, a meta-analysis found that propofol, alpha2-adrenoceptors, ketamine, fentanyl, and perioperative analgesia induce a prophylactic effect against emergence agitation [12]. However, the authors concluded that the analgesic properties of these treatments do not play a role and that reduced anesthetic requirements via potentiation of anesthesia might be involved [12]. Second, as another possible risk factor in this study, a younger age was associated with emergence agitation. In 1997, Aono et al. [13] showed a higher rate of emergence agitation in preschool boys anesthetized with sevoflurane compared with school boys. Since then, several studies confirmed that a young age was a factor associated with emergence agitation [14,15]. In pharmacodynamics, the minimal alveolar concentrations are very high in infants and decrease with increasing age [16]. In addition, in the very young subjects, blood solubility is reduced due to changing lipid and protein profiles, leading to a more rapid induction [16]. Thus, rapid awakening from sevoflurane in a younger age may partly explain the phenomenon, although rapid awakening is disputed [15,17].

In this study, 0.3 µg/kg of fentanyl was administered during the anesthetic induction. Although fentanyl as an adjuvant was reported to reduce the emergence agitation from sevoflurane anesthesia [18], the incidence of emergence agitation in this study was similar to that of Kim’s study (51% vs. 55%, respectively) [11]. First, fentanyl as a rescue analgesic in the PACU was administered immediately on arrival at the PACU without pre-assessment of the emergence agitation in Kim’s study. However, in this study, pain and emergence agitation scores were evaluated immediately on arrival at the PACU, and fentanyl administration was decided based upon the scores. The foregoing discussion suggests that the pain treatment in PACU does not influence the incidence of emergence agitation. Second, emergence agitation was rated using the PAED scale in this study compared with the Watch scale in Kim’s study. These two scales correlated reasonably well with each other, although the PAED scale showed greater a sensitivity in detecting emergence compared with the Watch scale [19].

In this study, the relationship between the nevus size and the postoperative pain varied when the two groups were compared or in the multivariate analysis. Only 38% of the pediatric patients receiving an excision for scalp nevi required rescue analgesics in the PACU, which was consistent with the results of the study by Xu et al. [5], in which a simple skin incision produced less severe and transient pain than an incision of the skin combined with deep tissue. In the development of pain, the extent of the deep tissue injury appears to be more meaningful than the length of skin excision. When two approaches with the same length of skin incision (20 cm) and different degrees of deep muscle tissue injury were compared, the approach entailing the incision of deep tissue induced greater pain at rest and following morphine use [20]. In contrast, when two approaches with different lengths of skin incision (9 vs. 16 cm) and the same degree of deep muscle tissue injury were compared, no difference was found in pains at rest and following morphine use [21]. However, a previous case reported unusual pain after a nevus excision, which transformed into a complex regional pain syndrome [22].

In an observational study with Turkish children, CMN showed differential localization according to the body region, predominantly in the head and neck or back [23]. During the study, a nevus excision in the facial area was suspected to involve greater pain and emergence agitation than in other areas; however, in a final analysis, body location did not influence the pain or agitation. Although quantification and comparison of pain according to body location is important, no consensus is available regarding the outcomes [24]. The sensitivity of the facial area to pain was found to be either higher [25] or similar to other body locations [26]. Distal parts were equally sensitive [27] or less sensitive than the proximal parts of the limb [25,26]. However, because the degree of sensitivity to noxious pain is uniform across the body, the comparison of pain sensitivity according to body location needs to be evaluated in a larger sample size.

## 5. Conclusions

A large-sized nevus triggered more severe emergence agitation in children following sevoflurane anesthesia; however, the nevus location had no effect on the pain or emergence agitation. In addition, a younger age was associated with pain and emergence agitation. Clinicians need to consider the proportion as a size parameter when managing pain and emergence agitation in children undergoing a nevus excision.

## Figures and Tables

**Table 1:** Nevus size and location in evaluating pain and agitation.

Variables		Pain			Agitation		
		Low (*n* = 50)	High (*n* = 50)	*p*-Value	Low (*n* = 50)	High (*n* = 50)	*p*-Value
Parameters for size	long diameter	3.07 ± 2.44	7.78 ± 17.59	0.066	3.10 ± 2.48	7.65 ± 17.43	0.071
	area before resection	4.10 ± 5.40	10.68 ± 17.76	0.015	5.34 ± 12.91	9.36 ± 13.83	0.137
	area of resection	3.39 ± 4.20	6.11 ± 7.35	0.026	3.33 ± 4.01	6.12 ± 7.38	0.021
	proportion	0.06 ± 0.08	0.11 ± 0.13	0.021	0.05 ± 0.07	0.11 ± 0.14	0.009
Nevus location	trunk	8 (16%)	3 (6%)	0.110	6 (12%)	5 (10%)	0.697
	face	26 (52%)	24 (48%)	0.689	26 (53%)	24 (47%)	0.548
	scalp	7 (14%)	13 (26%)	0.134	9 (18%)	11 (22%)	0.689
	extremities	9 (18%)	10 (20%)	0.799	8 (16%)	11 (22%)	0.504

Values are expressed as mean ± standard deviation or number (%). The proportion was defined as the ratio of nevi to the total body surface.

**Table 2:** Patient’s characteristics and operation details.

Variables	Small Group (*n* = 50)	Large Group (*n* = 50)	*p*-Value
Age (month)	34 (12–103)	31 (10–74)	0.338
Gender (M/F)	17/33	20/30	0.679
Height (cm)	93.4 (75–131)	92 (12.5–133)	0.242
Weight (kg)	14.5 (8.9–28)	13.8 (8–25)	0.471
BSA (cm^2^)	6120 (4460–10,094)	5879 (2946–9218)	0.275
Operation time (min)	15 (8–55)	30 (15–85)	<0.001
Anesthesia time (min)	55 (15–110)	67.5 (20–115)	<0.001
Long diameter (cm)	1.7 (0.5–11.7)	4.7 (0.5–93)	<0.001
Area before resection (cm^2^)	0.95 (0.25–3.25)	6.75 (2.5–88.5)	<0.001
Area of resection (cm^2^)	0.875 (0.25–3.25)	5.625 (2.25–33.5)	<0.001
Proportion (%)	0.014 (0.003–0.041)	0.097 (0.043–0.560)	<0.001

Values are expressed as median (min–max) or number (%); proportion was defined as the ratio of nevi to the total body surface; M, male; F, female; BSA, body surface area.

**Table 3:** PACU data.

Variables	Small Group (*n* = 50)	Large Group (*n* = 50)	*p*-Value
Highest pain score	5.5 (0–10)	7.5 (0–10)	0.057
Highest agitation score	6 (0–20)	12.5 (0–20)	0.021
Vomiting	0	2 (4%)	0.495
Patients receiving analgesic	26 (52%)	21 (41%)	0.423
Duration of PACU stay (min)	60 (30–110)	62.5 (30–120)	0.258

Values are expressed as median (min–max) or number (%); PACU, postanesthesia care unit.

**Table 4:** The multivariate linear regression analysis predicting pain and agitation after a nevus excision.

Variables		*β*	SE	*p*-Value
Pain	age	−0.049	0.014	0.001
	proportion	7.913	2.709	0.004
Agitation	age	−0.097	0.031	0.002
	proportion	15.803	6.061	0.011

The proportion was defined as the ratio of nevi to total body surface; *β*, logistic regression coefficient; SE, standard error.
